# Nivolumab Hypersensitivity Reactions a Myth or Reality in Solid Tumors—A Systematic Review of the Literature

**DOI:** 10.3390/curroncol29120741

**Published:** 2022-12-02

**Authors:** Cristina-Florina Pîrlog, Andreea Ioana Paroșanu, Cristina Orlov Slavu, Mihaela Olaru, Ana Maria Popa, Cristian Iaciu, Irina Niță, Pompilia Moțatu, Cotan Horia, Loredana Sabina Cornelia Manolescu, Cornelia Nițipir

**Affiliations:** 1 Department of Medical Oncology, Elias Emergency University Hospital, 011461 Bucharest, Romania; 2 Department of Oncology, Faculty of Medicine, “Carol Davila” University of Medicine and Pharmacy, 050474 Bucharest, Romania; 3 Department of Medical Oncology, Monza Oncology Hospital, 013821 Bucharest, Romania; 4 Department of Medical Oncology, Municipal Hospital Ploiesti, 100409 Ploiesti, Romania; 5 Department of Microbiology, Parasitology and Virology, Faculty of Midwifery and Nursing, “Carol Davila” University of Medicine and Pharmacy, 050474 Bucharest, Romania; 6 Department of Virology, Institute of Virology “Stefan S. Nicolau”, 030304 Bucharest, Romania

**Keywords:** Nivolumab, hypersensitivity reaction, immune-checkpoint inhibitors

## Abstract

Immune-checkpoint inhibitors (ICIs) are the most effective treatments nowadays. Nivolumab was the second ICI used for treating solid tumors with amazing results. Patients treated with Nivolumab may react differently to this treatment. Some people tolerate this treatment very well without experiencing any adverse reactions, whilst some may have mild symptoms and a part of them can present severe reactions. In our research, we sought to identify the answers to four questions: 1. what type of cancer has more severe hypersensitivity reactions to Nivolumab, 2. what is the time frame for developing these severe reactions to Nivolumab, 3. whether it is best to continue or stop the treatment after a severe hypersensitivity reaction to Nivolumab and 4. what severe hypersensitivity reactions are the most frequent reported along Nivolumab treatment. This review also highlights another problem with regard to the usage of concomitant and prior medications or other methods of treatment (e.g., radiation therapy), which can also lead to severe reactions. Treatment with Nivolumab is very well tolerated, but patients should also be warned of the possibility of severe hypersensitivity reactions for which they should urgently see a doctor for a personalized evaluation. There are some options for individuals with severe hypersensitivity reactions, for eg. switching the medication or applying a desensitization protocol.

## 1. Introduction

In recent years, immunotherapy has become the most used treatment in metastatic cancers besides targeted treatments. Immune-checkpoint inhibitors (ICIs) are molecules that enhance the activity of the immune system by blocking the interaction between programmed cell death 1 (PD-1) and its ligand: programmed cell death ligand 1 (PD-L1) and programmed cell death ligand 2 (PD-L2); or by blocking the costimulatory molecule represented by cytotoxic T lymphocyte-associated protein 4 (CTLA-4) [1,2,3,4,5]. They are divided into 3 classes: PD-1 inhibitors (Nivolumab, Pembrolizumab, Cemiplimab, and Dostarlimab), PD-L1 inhibitors (Avelumab, Atezolizumab, and Durvalumab) and CTLA-4 inhibitors (Ipilimumab).

Nivolumab, a fully human IgG 4 antibody, directed against PD-1, is the first ICI treatment approved for metastatic lung cancer [5,6,7]. It is generally very well tolerated by patients, but can induce immune-related adverse effects (irAEs), which could affect any organ system. The adverse reactions consist of endocrine reactions (e.g., hypothyroidism, hyperthyroidism, thyroiditis), skin reactions (e.g., rash, pruritus, vitiligo, dry skin, erythema, urticaria), metabolic reactions (e.g., decreased appetite, hyperglycemia, hypoglycemia), pulmonary reactions (e.g., dyspnea, cough, pneumonitis, pleural effusion), gastrointestinal reactions (e.g., diarrhea, abdominal pain, constipation, colitis, stomatitis, dry mouth), cardiac reactions (e.g., tachycardia, atrial fibrillation, myocarditis), neurologic reactions (e.g., headache, peripheral neuropathy, dizziness), blood disorders (e.g., lymphopenia, anemia, leucopenia, neutropenia, thrombocytopenia), fatigue, pyrexia, oedema [8]. The hypersensitivity type of reactions to Nivolumab is very rare, between 1% and 3% [9,10], but it is important for them to be recognized early because they need prompt intervention.

A hypersensitivity reaction is an inappropriate or exaggerated immune response to either an antigen or allergen. The hypersensitivity reactions can be divided into two subgroups of immediate reactions (<1 h) or delayed reactions (>1 h) [11]. Immediate reactions are type I and include pruritus, edema, urticaria, and anaphylactic shock [12]. The delayed ones are type IV reactions, mediated by T-cells, and include drug-induced agranulocytosis (DIA), drug-induced skin disorders (DISI), drug-induced liver injury (DILI), and drug-induced renal injury (DIRI) [11]. The DISI group contains contact allergy, fixed drug eruption (FDE), acute, generalized exanthematic pustulosis (AGEP), maculopapular rash (MPR), Stevens–Johnson Syndrome (SJS), toxic epidermal necrolysis (TEN), drug reaction with eosinophilia and systemic symptoms (DRESS) [11,13]. The hypersensitivity reactions may be regarded as a subcategory of irAEs in patients receiving ICIs [14].

## 2. Materials and Methods

This review has been conducted meeting the guidelines of Preferred Reporting Items for Systematic Reviews (PRISMA 2020). The registration number: no 41482 and the date of approval is 9 August 2022.

### 2.1. The Review Questions

Are there many severe hypersensitivity reactions to Nivolumab described in the literature? What type of cancer has the most severe hypersensitivity reactions to Nivolumab? What is the time frame for developing severe hypersensitivity reactions to Nivolumab? How are Nivolumab hypersensitivity reactions managed through the literature search? What types of severe hypersensitivity reactions are more frequently reported?

### 2.2. Literature Search

The literature search was performed in the electronic database of PubMed, and the following combination has been researched: “Nivolumab hypersensitivity reaction”. The search was performed between 2013 and September 2022. The relevant articles related to the questions of this review were identified and only those that met the eligibility criteria were assessed. The inclusion and exclusion criteria are provided in Table 1.

### 2.3. Data Extraction and Presentation

**Figure 1:**
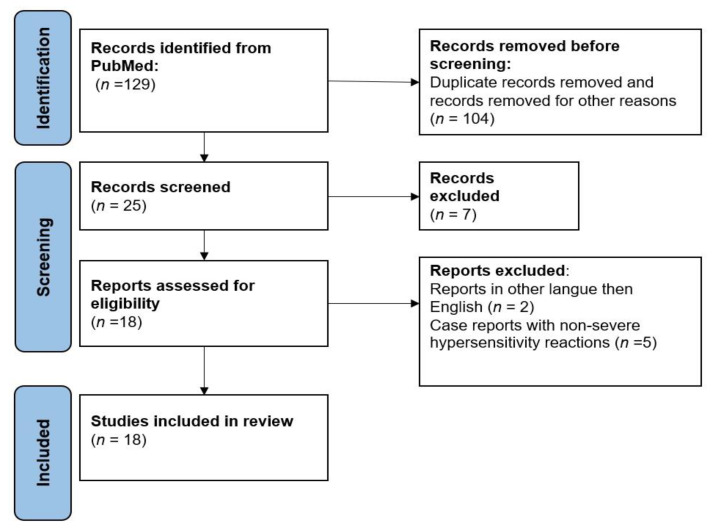
Flow chart showing the strategy used in this systematic review.

Using the aforementioned combination of keywords, we searched for the articles published in PubMed. The search yielded a total of 129 articles, of which 104 of them were irrelevant or duplicated. Among the 25 articles relevant to this review, only 18 studies met the eligibility criteria and were included in this systematic review. The flow chart of the research is presented in Figure 1.

## 3. Results

This research included only 18 articles. These articles are case reports of severe hypersensitivity reactions to Nivolumab. We excluded from this study the cases with non-severe reactions, grade 1 or 2 according to CTCAE v 5.0, November 2017 [15], and erythema nodosum, lichen planus, morphea, lichen sclerosus et atrophicus. All the included studies are summarized in Table 2.

## 4. Discussion

Among the irAEs of ICIs, the most frequently reported are the dermatological ones, which consist of rash and pruritus. They are present in up to 25% of melanoma patients and in 10% or more of non-small-cell lung cancer patients [33,34,35]. Other dermatological irAE (dirAEs) are vitiligo-like depigmentation, morbilliform exanthem, lichenoid dermatitis, bullous pemphigoid, and severe cutaneous irAEs like Stevens-Johnson syndrome (SJS)/toxic epidermal necrolysis (TEN), acute generalized exanthematous pustulosis (AGEP) and drug reaction with eosinophilia and systemic symptoms (DRESS). Xerosis, alopecia areata, stomatitis, urticaria, photosensitivity reactions [9,17], hyperhidrosis, skin exfoliation [34,36], and hair color changes are rare, but present in daily practice [37].

Most of the dirAEs are low to moderate grades according to the CTCAE v 5.0, November 2017 [15]. In grade 1 and 2 dirAEs, the treatment consists of topical corticosteroids with moderate or high potency and supportive care. Severe dirAEs (grade 3 and grade 4) require the administration of high-dose systemic corticosteroid treatment, intense supportive care management, and wound management. In some cases, it is a must to administer tumor necrosis factor alpha (TNF-α) inhibitors (Infliximab or Etanercept), Mycophenolate mofetil, Cyclosporin, and even plasmapheresis [34,36] These reactions require a definitive interruption of immunotherapy [38,39,40].

In our study, we found nineteen cases of severe hypersensitivity reactions to Nivolumab: nine cases of female patients, seven cases of male patients, and three cases without the sex of the patient mentioned in the article. Regarding the solid tumor’s distribution, there were 6/19 patients with gastrointestinal cancers (31.58%), 5/19 lung cancer patients (26.32%), 4/19 melanoma patients (21.05%), 3/19 renal cancer patients (15.79%) and 1/19 patients with head and neck cancer (5.26%) who have developed severe hypersensitivity reactions to Nivolumab. It appears that Nivolumab hypersensitivity reactions are more frequent in patients with gastrointestinal cancers. This may be due to the fact that the gut microbiome is considered responsible for the modulation of immune responses. Lu Y highlighted in his review that the gut microbiome can generate increased responses to ICIs by adjusting the CD8+ T cells, T helper 1, and tumor-associated myeloid cell proportions [41]. In a study of fecal microbial transplantation, the mouse who received the transplant had an increased level of CD8+ T cells [42]. In another review article, Pierrard J. et al. showed that the development of irAEs in mouse models can be influenced by the composition and modifications in the gut microbiome [43]. Patients with Bifidobacterium longum, Collinsella aerofaciens and Enterococcus faecium present in gut microbiome show good responses to anti-PD-1 therapy. In some studies, the Ruminococaceae family of the Firmicutes phylum is responsible for the development of irAEs [44,45].

Most of the hypersensitivity reactions were present in the first week after starting the treatment with Nivolumab and only four of the authors reported different times for the development of the adverse reactions. P. Basu et al. reported the presence of TEN after 2 years [17], Constantin A Dasanu et al. reported the presence of SJS/TEN at 16 weeks [18], Yi-Tsz Lin et al. reported the presence of SJS after 18 cycles [23], while Yuko Watanabe et al. reported them at 7 months [25].

In recent studies, the efficacy of ICIs has been related to the development of irAE in NSCLC patients [45,46]. In NSCLC patients it has been demonstrated by Hussaini et al. that patients with irAE have a better objective response rate (ORR) (41.49% vs. 18.01% without irAEs), a better progression-free survival (PFS) (median 8.97 vs. 3.06 months) and a longer overall survival (OS) (median 19.07 vs. 7.45 months) [47,48]. In melanoma patients, the development of dirAE, especially vitiligo, is associated with the efficacy of ICI treatment in stages III and IV [49,50,51]. Other reports, sustain that the development of irAE was associated with a higher objective response rate (ORR), but not with progression-free survival (PFS) [52].

It is very essential to know the patient previous medications used to treat their cancers or if the patient was exposed to radiation therapy. We should check this information on the hospital networks or hospital records. In our research, five authors reported severe hypersensitivity reactions after Nivolumab was stopped. Yasushige Takeda et al. documented a case of SJS after two cycles of S-1 and Docetaxel for gastric cancer, the author mentioned that the patient received Nivolumab before the start of the first cycle of S-1 and Docetaxel [20]. Takayoshi Komatsu-Fujii et al. reported a case of SJS in a patient receiving tegafur/gimeracil/oteracil (TS-1) who was previously treated with Nivolumab for his disease [53]. Yi-Tsz Lin et al. showed a case of SJS induced by treatment with Esomeprazole in a patient who received Nivolumab for advanced lung cancer [23]. M Arenbergerova et al. related a case of TEN induced by Vemurafenib after discontinuation of Nivolumab [54]. Maximova N et al. presented another patient diagnosed with DRESS induced by Vemurafenib administration after Nivolumab, who responded very well to the administration of Tocilizumab and Infliximab [29]. Also, there are some reports of SJS syndrome induced by radiation in patients receiving Nivolumab [24,55] or anticonvulsant drugs like phenytoin [56], phenobarbital [57], and amifostine [58].

Only two cases in our research of the literature continued desensitization to Nivolumab as an alternative to continuing therapy: a 57 years old female with kidney cancer who developed an infusion reaction to Nivolumab during her third cycle and another patient with kidney cancer [22,31]. In one of the 19 cases Nivolumab was switched with Pembrolizumab and in another one, the combination of Vemurafenib and Cobimetinib was changed to Trametinib and Dabrafenib. The patients stopped showing signs of adverse reactions [16,28]. The combination of chemotherapy, antibiotics and antiretroviral therapy brings more adverse reactions than the treatment with just Nivolumab [45,59,60,61].

Although our research has only a limited number of studies, the distribution of the severe hypersensitivity reactions to Nivolumab was the following: 42.10% of the patients developed SJS (8/19 patients), 26.32% of the patients developed TEN (5/19 patients), 15.79% of the patients developed infusion reactions (3/19 patients), 10.53%of the patients developed DRESS (2/19 patients) and 5.26% of the patients developed CCR grade 3 (1/19 patients). This is the first study reporting the distribution of severe hypersensitivity reactions on Nivolumab in solid tumors.

In our literature research, 10.53% of the patients (2/19 patients) died of the complications of the hypersensitivity reactions and 15.79% of the patients (3/19 patients) presented progression to disease and died afterward [17,19,23,25,26]. Only 5.26% of the patients (1/19 patients) had a complete response to Nivolumab treatment after DRESS and 15.79% of the patients (3/19 patients) had stable disease [9,22,28,31].

Our review has some limitations. We could include in our study only 18 articles on severe hypersensitivity reactions to Nivolumab. We focused our search only on solid tumors and did not include hematological malignancies. Furthermore, our findings need to be extended to include the hematological malignancies treated with Nivolumab.

## 5. Conclusions

The treatment with Nivolumab is very effective and very well tolerated by most patients without developing any irAE. It is crucial to recognize the prodromal symptoms of severe reactions to the Nivolumab treatment and to promptly intervene. As our research of the literature showed, switching the medication or applying a desensitization protocol are options for severe reactions to Nivolumab. 

## Figures and Tables

**Table 1:** Inclusion and exclusion criteria.

	Inclusion Criteria	Exclusion Criteria
1	Any article published in English or translated into English.	Any article or case report that was incomplete or in other languages.
2	Articles published between 2013 and September 2022.	Any article or case report that included non-severe hypersensitivity reactions, grade 1 or 2, according to Common Terminology Criteria for Adverse Events (CTCAE v 5.0, November 2017) [15] in solid tumors and erythema nodosum, lichen planus, morphea, lichen sclerosus et atrophicus.
3	Case reports of Nivolumab hypersensitivity reactions type I and type IV in solid tumors, grade 3 or 4 according to the Common Terminology Criteria for Adverse Events (CTCAE v 5.0, November 2017) [15]	

**Table 2:** Cases of Nivolumab hypersensitivity reactions.

	Reference/Jornal	Drug Type	Type of Reaction	Age (Years)/ Sex	Type of Cancer	Time to Appearance	Prognosis/ Treatment
1	Briana Choi et al./Am J Health Syst Pharm 2019 [16]	Nivolumab	Infusion reaction	70/ male	Hepatocarcinoma	At the 2nd dose of Nivolumab	The patient switches the medication to Pembrolizumab.
2	Luoyan Ai et al./J Immunother Cancer 2021 [9]	Nivolumab	DRESS	72/ male	Stage III/Gastric cancer	3 weeks after Nivolumab initiation	The patient had a full course of steroid therapy. He had a complete response to Nivolumab treatment.
3	P Basu et al./British Journal of Dermatology 2020 [17]	Nivolumab	TEN	50/ female	Metastatic colon cancer	After 2 years	The patient died due to TEN.
4	Constantin A Dasanu et al./ J Oncol Pharm Pract. 2019 [18]	Nivolumab	SJS/TEN	-	Hepatocarcinoma	16 weeks	-
5	Tamara Gracia-Cazaña et al./Dermatol Online J 2021 [19]	Nivolumab	SJS	78/ male	Metastatic lung cancer	After the 2nd cycle	The patient died from disease progression 16 weeks after his discharge.
6	Yasushige Takeda et al./Gan to Kagaku Ryoho 2021 [20]	Nivolumab	SJS	51/ male,	Metastatic gastric cancer	After the 2nd cycle of S1 and Docetaxel	-
		Nivolumab	SJS	75/ female	Metastatic gastric cancer	After 1st cycle of Nivolumab	-
7	M Salati et al./ Ann Oncol 2018 [21]	Nivolumab	SJS	59/ female	Metastatic lung cancer	After the 2ndcycle	The patient was discharged after 15 days of hospitalization.
8	Laura Sánchez Togneri et al./J Oncol Pharm Pract 2022 [22]	Nivolumab	cytokine release reaction (CRR)-grade 3	-	Metastatic renal cell cancer	-	The patient had a desensitization protocol with specific premedication and was stable in his disease.
9	Yi-Tsz Lin et al./Lung Cancer 2020 [23]	Nivolumab	SJS	52/ female	Metastatic lung cancer	After the 18th cycle	The patient recovered from SJS, but died from the progression of her disease.
10	Kishan M Shah et al./Dermatol Online J 2018 [24]	Nivolumab	SJS	63/ male	Head and neck cancer	After the 1st cycle	The patient recovered, but treatment with Nivolumab was discontinued permanently.
11	Yuko Watanabe et al./Eur J Cancer 2020 [25]	Nivolumab	TEN	60/ female	Advanced melanoma	At 7 months after initiation of treatment (1 month after discontinuation)	The patient died of melanoma progression 3 months after the onset of TEN.
12	Karina L Vivar et al./J Cutan Pathol 2017 [26]	Nivolumab	TEN	-	Metastatic melanoma	-	The patient died of this complication.
13	Namrata Nayar et al./J Immunother 2016 [27]	Nivolumab	TEN	64/ female	Metastatic melanoma	After the 2nd cycle	-
14	Naotaka Kumada et al./Hinyokika Kiyo 2022 [28]	Nivolumab	SJS	65/ male	Metastatic renal cancer	On day 28 after the 1st cycle	6 months after discharge, the patient had stable disease.
15	Natalia Maximova et al./ J Immunother Cancer 2020 [29]	Nivolumab	DRESS	41/ female	Melanoma	On day 4 after initiation of Vemurafenib and Cobimetinib, after four cycles of Nivolumab	Treatment was changed with Dabrafenib and Trametinib. 1 month after discharge, the patient showed no signs of drug toxicity.
16	Seema Kumari et al./ Cancer Reports 2020 [30]	Nivolumab	Infusion reaction	61/ male	Metastatic lung cancer	At the 2nd cycle	The patient was discharged, but died of Influenza A.
17	Sebastián Ramírez-Cruz et al./Farmacia Hospitalaria 2020 [31]	Nivolumab	Infusion reaction	57/ female	Metastatic renal cancer	At the 3rd cycle	The patient had stable disease and continued Nivolumab with a desensitization protocol.
18	Jiro Ito et al./Lung Cancer 2017 [32]	Nivolumab	SJS, severe pruritus	76/ female	Advanced lung cancer	After the 2nd cycle	The patient had a progressive disease.

## Data Availability

Not applicable.
